# Photoluminescence Properties of Cyan-Emitting Lu_3_Ga_x_Al_5−x_O_12_: Ce^3+^ Garnet Phosphors Synthesized in Nonreducing Atmosphere and at Different Temperature for High Quality w-LEDs

**DOI:** 10.3390/ma15196817

**Published:** 2022-09-30

**Authors:** Ming-Yang Qu, Ting-Qu Li, Qiao-Li Liu

**Affiliations:** 1 Key Laboratory for Special Functional Materials in Jilin Provincial Universities, Jilin Institute of Chemical Technology, Jilin 132022, China; 2 School of Materials Science and Engineering, Key Laboratory of Mobile Materials, MOE, and State Key Laboratory of Superhard Materials, Jilin University, Changchun 130012, China

**Keywords:** cyan phosphor, photoluminescence, band gap, thermal ionization

## Abstract

The existence of so-called blue-green cavities in the luminescence spectrum has been a hindrance to the improvement in the performance of traditional phosphor-converted white light emitting diodes. The commercial phosphors synthesized in reducing atmospheres can also cause problems such as equipment complexity, increased cost, and environmental pollution. Herein, a series of cyan-emitting Lu_3_Ga_x_Al_5−x_O_12_: Ce^3+^ (x = 0, 1, 2, 3, 4) garnet phosphors were synthesized by a traditional solid-state reaction in a nonreducing atmosphere at different temperatures. The crystal structure, grain morphology, optical properties, and thermal quenching behavior were used to analyze the optical properties of the as-prepared phosphors. The luminescence intensity of samples is affected by the synthesis temperature and energy gap between the conduction band and the lowest energy of the 5d excited state of the host lattice. With the substitution of Al^3+^ by Ga^3+^, the regularity of the excitation and emission band movement is determined by the combined effects of crystal field splitting (CFS) and the nephelauxetic effect (NE). The temperature dependence of luminescence was studied. The thermal quenching mechanism was clarified by the thermal ionization model. Finally, by employing Lu_2.94_Ga_2_Al_3_O_12_: Ce^3+^_0.06_ as a cyan component, a w-LED with a high color rendering index of 93.2 and low correlation color temperature of 3880 K based on a blue chip and commercial red phosphors were fabricated in order to explore its possible application in high quality w-LED.

## 1. Introduction

White light emitting diodes (w-LEDs) are becoming a new generation of solid-state light sources for the lighting industry and display systems due to their excellent properties such as energy-saving, high energy efficiency, environment friendly, and small volume [1,2,3,4,5,6]. Nowadays, the main challenge for w-LEDs is to actualize high luminous efficiency, outstanding color rendering properties, moderate correlated color temperature, and excellent resistance to the thermal quenching behavior of photoluminescence. The most common way to realize white light via w-LEDs is composed of a blue LED chip and the yellow-emitting phosphors of Y_3_Al_5_O_12_: Ce^3+^ (YAG: Ce^3+^). However, this kind of LED suffers from limitations in the lack of red- (580–650 nm) and green-emission (470–510 nm) components in the white light, resulting in a poor color rendering index (CRI) and harsher correlation color temperature (CCT) [7,8]. Thus, the demand for indoor lighting and operating room lighting cannot be met. To solve this problem, appropriate red phosphors were added into a yellow-emitting phosphor, and even green phosphor and red phosphor were mixed to replace the yellow one to improve CRI and depress CCT.

For green phosphor, one of the necessary tasks is to develop cyan-emitting phosphors with peak values in the 480–500 nm emission region so that the blue-green cavity around 490 nm in the emission spectrum can be filled to adapt for high-quality w-LEDs. Up to now, many green phosphors have been developed to achieve this goal such as La_3_Br(SiS_4_)_2_: Ce^3+^, BaSi_7_N_10_: Eu^2+^, and Ca_2_LaZr_2_Ga_3_O_12_: Ce^3+^ [9,10,11]. Among them, Y_3-y_Al_5−x_Ga_x_O_12_: yCe^3+^ (YAGG: Ce^3+^) is particularly noteworthy. It has been reported that the Ga^3+^ substituted YAG: Ce^3+^ phosphor can achieve a tunable color from yellow- to green-emitting, which implies that Ga^3+^ can achieve a spectral blue shift. In past studies, many researchers have investigated the structural transformation and luminescence properties of YAGG: Ce^3+^ such as the preferential entry of Ga^3+^ ions into different lattices of matrix [12,13], and the effect of Ga^3+^ ion content on its luminescence properties at different temperature [14,15,16,17]. However, it should also be noted that these phosphors have their own defects, for instance, the emission wavelength of Y_3_Al_5−x_Ga_x_O_12_: Ce^3+^ can be blue shifted to 500 nm with x = 4, but the emission intensity decreases significantly; there is poor chemical stability and a high thermal quenching for La_3_Br(SiS_4_)_2_: Ce^3+^ with the emission peak at 466 nm based on near-ultraviolet (n-UV) LED chip; an expensive and harsh synthetic process for BaSi_7_N_10_: Eu^2+^; and a low quantum efficiency for Ca_2_LaZr_2_Ga_3_O_12_: Ce^3+^. Hence, it is still necessary to develop blue-green cyan phosphors aimed at improving the color rendering properties and thermal stability of luminescence, expanding the application field of blue-green phosphor for w-LEDs. As a kind of green-emitting phosphor with an emission wavelength around 510 nm, Lu_3_Al_5_O_12_: Ce^3+^ (LuAG: Ce^3+^), synthesized in a reducing atmosphere, has always been a matter of great concern because of its excellent luminescence efficiency and high thermal stability [18,19]. However, its emission spectrum is still not sufficient to fill the cavity near 490 nm. Therefore, it needs to be modified to achieve a further spectral blue-shift to meet the requirements.

Moreover, as known to all, the process of synthesis in a nonreducing atmosphere has numerous advantages such as a simple process, low cost, non-toxic, and so on. In the synthesis process of Y_2.94_Al_3_Ga_2_O_12_: 0.06Ce^3+^ reported by Zhu et al., it was found that sintering under a reducing atmosphere would lead to the appearance of Ga_2_O_3_, resulting in a hindrance in forming a pure phase of YAGG: Ce^3+^. It also indicated that the structure and properties of the material would be greatly affected by the synthetic atmosphere [20]. However, the luminescence properties of Ce^3+^ doped Lu_3_Ga_x_Al_5−x_O_12_ garnet-typed phosphors synthesized in a nonreducing atmosphere have not been especially reported. Meanwhile, the influence of the synthesis temperature on the luminescence intensity has also not been involved in the previous reports of these phosphor systems. In order to narrow the cyan gap in the spectrum and improve the photoluminescence performances of the w-LEDs device, herein, we synthesized a series of excellent blue-light excited cyan phosphor Lu_2.94_Ga_x_Al_5−x_O_12_: 0.06Ce^3+^ (LuAGG: Ce^3+^) based on the substitute of Al^3+^ by Ga^3+^ in the host. The effects of the synthesis conditions and concentration of Ga^3+^ ions on the luminescence and thermal stability of the samples were investigated systematically. Finally, a w-LED device was fabricated by using a blue GaN chip with the as-prepared cyan phosphor Lu_2.94_Ga_2_Al_3_O_12_: Ce^3+^_0.06_ and commercial red phosphor Sr_2_Si_5_N_8_: Eu^2+^, which has an excellent CRI of 93.2 at a CCT of 3880 K, indicating its identity of a promising candidate for high quality w-LEDs.

## 2. Experimental Section

### 2.1. Materials and Synthesis

A nominal formula of Lu_2.94_Ga_x_Al_5−x_O_12_ (x = 0, 1, 2, 3, 4) doped with 0.06Ce^3+^, stoichiometric amounts of Al_2_O_3_ (99%, Ganzhou Kemingrui Nonferrous Metals Co. Ltd., Ganzhou, China), Lu_2_O_3_ (99.9%, Ganzhou Kemingrui Nonferrous Metals Co. Ltd., China), Ga_2_O_3_ (99%, A.R. Shanghai Macklin Biochemical Co. Ltd., Shanghai, China), Ce(NO_3_)_3_·6H_2_O (99%, A.R. Shanghai Macklin Biochemical Co. Ltd., Shanghai, China), and H_3_BO_3_ (99%, A.R. Tianjin Guangfu Co., Ltd., Tianjin, China) as a flux (4%) was weighed and prepared for mixing in an agate mortar for 0.5 h. The mixtures were placed in the alumina crucible in an airtight atmosphere at different temperatures (1350 °C, 1450 °C, and 1550 °C for 3 h). Finally, the products were naturally cooled to room temperature for complete reaction, and ground into powder for further analysis.

### 2.2. Characterization

The crystallographic structure of the samples was detected by powder X-ray diffraction (XRD, Rigaku, Ultima IV, Tokyo, Japan) using the parameters of 40 kV and 20 mA, and continuous scanning with the scanning speed of 4°/min was implemented in the scanning range of 10–80°. The emission and excitation spectra (PL and PLE) were monitored via a fluorescence spectrophotometer (model F-7000, Hitachi Ltd., Tokyo, Japan) equipped with a 150 W Xe lamp. The field-emission scanning electron microscope (SEM, JSM-6701F, JEOL, Akishima, Japan) was used to observe the gain morphology of the as-prepared samples at voltages of 3 kV. The temperature-dependence of the PL spectra were also recorded by the F-7000 fluorescence spectrometer attached to the homemade temperature controller at the range of 30–225 °C. Finally, a w-LED specimen was assembled with commercial red phosphors (Sr_2_Si_5_N_8_: Eu^2+^), a representative sample of Lu_2.94_Ga_2_Al_3_O_12_: Ce^3+^_0.06_ and GaN chips (λ_em_ = 450 nm). Its luminescence properties were evaluated on a SENSING SL-300 spectroradiometer. Except for the temperature-dependence of PL spectra, all tests were carried out at room temperature.

## 3. Results and Discussions

### 3.1. Crystal Structure Analysis and Micro-Morphology

**Figure 1:**
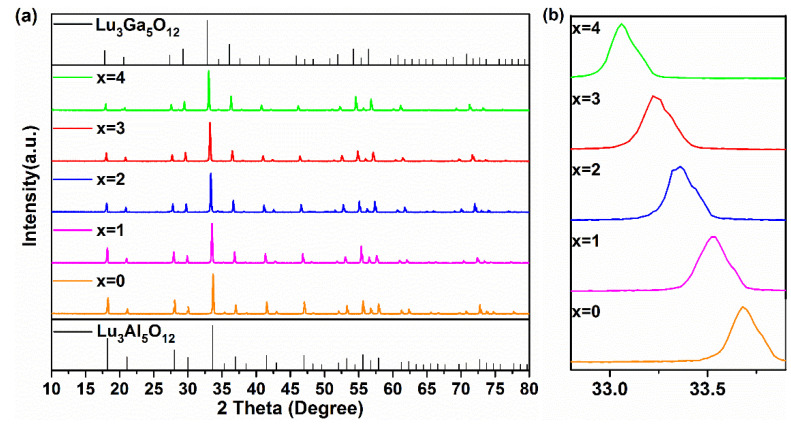
(**a**) The XRD patterns and (**b**) enlarged XRD patterns around 33.5° of the Lu_2.94_Ga_x_Al_5−x_O_12_: 0.06Ce^3+^ (x = 0–4) phosphors at 1450 °C for 3 h.

Figure 1a presents the XRD patterns of Lu_2.94_Ga_x_Al_5−x_O_12_: 0.06Ce^3+^ (x = 0, 1, 2, 3, 4) synthesized at 1450 °C. All of these samples exhibited almost the same XRD patterns to that of the Lu_3_Al_5_O_12_ (LuAG, PDF#73-1368) pure phase. Since no impurities with other diffraction peaks were found in the figure, this indicated that the pure phase of these compounds could be successfully prepared due to the combined effect of the sintering process at 1450 °C/3 h (3 h) and co-solvents. With the increase in the Ga^3+^ content, the LuAGG: Ce^3+^ structure gradually evolved from Lu_3_Al_5_O_12_ to Lu_3_Ga_5_O_12_ (LuGG, Ga-PDF#73-1372).

The detailed XRD patterns from 32.8° to 33.8° are depicted in Figure 1b. It is clear that the enlarged diffraction peaks belong to the (4 2 0) crystal face of the sample shifted to lower angles in the process of the increasing replacement of Ga^3+^ for Al^3+^ in the host, demonstrating that Ga_(1)_^3+^ (62 pm) and Ga_(2)_^3+^ (47 pm) had a bigger cationic radius instead of Al_(1)_^3+^ (53.5 pm), and Al_(2)_^3+^ (39 pm) entered the matrix of LuAG, resulting in lattice expansion.

**Figure 2:**
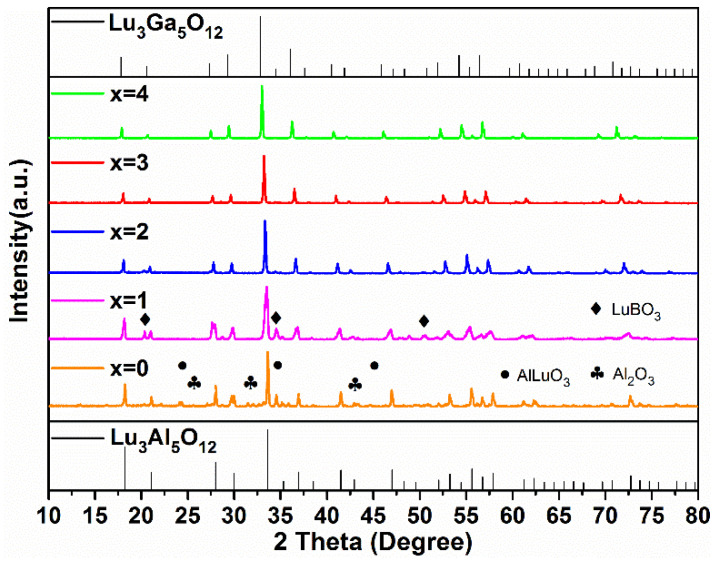
The XRD patterns of the Lu_2.94_Ga_x_Al_5−x_O_12_: 0.06Ce^3+^ (x = 0–4) phosphors synthesized at 1350 °C for 3 h.

In order to further explore the possibilities of low energy consumption synthetic processes, the Lu_2.94_Ga_x_Al_5−x_O_12_: 0.06Ce^3+^ samples with different Ga^3+^ concentrations (x = 0, 1, 2, 3, 4) were synthesized at 1350 °C for 3 h, whose XRD patterns are shown in Figure 2.

As x ≥ 2, there were no diffraction peaks of the impurity phase in the XRD patterns. However, as x is less than 2, diffraction peaks of Al_2_O_3_ (PDF#46-1215, 74-1081), AlLuO_3_ (PDF#24-0690), and LuBO_3_ (PDF#74-1938) were found in the Lu_2.94_Al_5_O_12_: 0.06Ce^3+^ and Lu_2.94_GaAl_4_O_12_: 0.06Ce^3+^ phosphors. It is clearly indicated in the figure that the higher the Ga^3+^ concentration, the lower the formation temperature of the pure phase LuAGG becomes. The reason for this phenomenon can be explained by the bond dissociation energies. The value of the Ga-O pair (285 kJ/mol) was obviously less than that of the Al-O pair (512 kJ/mol). It can be concluded that Ga^3+^ ions are easier to combine with O^2−^ ions, promoting the formation of a garnet structure at lower temperature. In contrast, when Al^3+^ ions completely replace Ga^3+^ in the raw material, a higher temperature is required to synthesize the Lu_3_Al_5_O_12_ pure phase.

**Figure 3:**
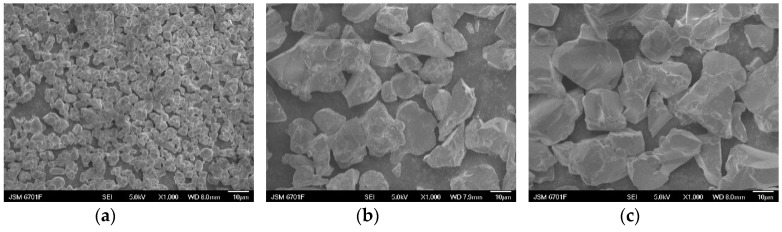
The SEM images of the Lu_3_Ga_2_Al_3_O_12_: Ce^3+^ synthesized at (**a**) 1350 °C, (**b**) 1450 °C, and (**c**) 1550 °C for 3 h.

It is well-known that the size, crystallinity, and morphology of phosphor can affect its emission intensity. In this case, the SEM images of the Lu_2.94_Ga_2_Al_3_O_12_: 0.06Ce^3+^ phosphors sintered at 1350 °C, 1450 °C, and 1550 °C were exhibited in Figure 3. It can be observed from the figure that, as the temperature rises, the diameter range of the aggregated particle increased from 2–5 to 20–30 μm, and the morphology of the grain changed from an oval shape to an irregular shape. The reason for this phenomenon can be attributed to the increasing concentration of Ga^3+^ ions contributing to the decrease in the phase formation temperature. Furthermore, the size of the phosphor particles gradually grew with the increasing sintering temperature, and the sample shrank more seriously. Finally, it resulted in an increased hardness of the samples and led to fragmented crystal grains after grinding.

### 3.2. Photoluminescent Spectra

**Figure 4:**
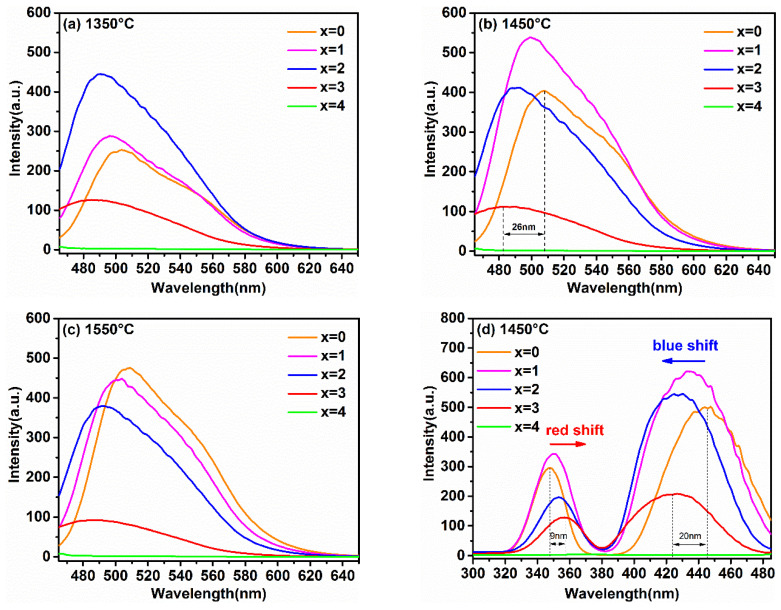

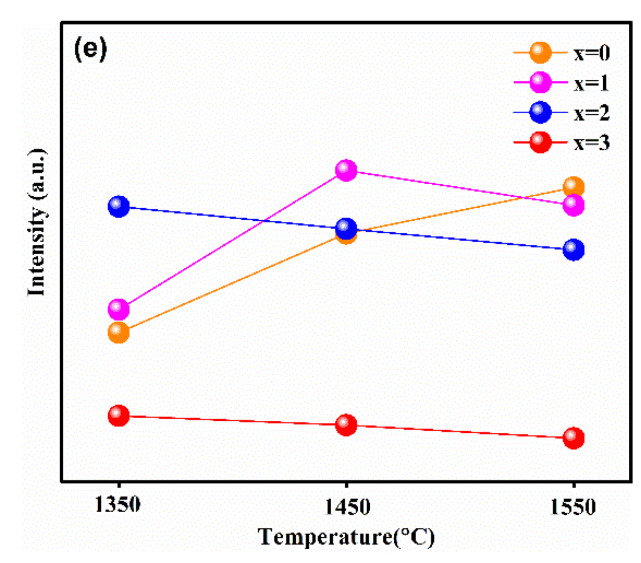
The emission spectra (λ_ex_ = 450 nm) of Lu_3_Ga_x_Al_5−x_O_12_: Ce^3+^ (x = 0, 1, 2, 3, 4) at (**a**) 1350 °C, (**b**) 1450 °C, (**c**) 1550 °C, (**d**) the excitation spectra (λ_em_ = 500 nm) of samples synthesized at 1450 °C and (**e**) the relationship of the maximum emission intensity versus the sintering temperature at different Ga^3+^ concentrations.

In order to reveal the relationship between the luminescence properties, the concentration of Ga^3+^ ions, and synthesis temperature, the PL and PLE spectra of the samples synthesized at different temperatures are recorded in Figure 4a–e. Figure 4e shows the relationship between the maximum emission intensity and sintering temperature at different Ga^3+^ concentrations. It can obviously be observed that the variation trend in the maximum emission intensity of samples with different Ga^3+^ concentrations with the increasing temperature was different. Furthermore, when the synthetic temperature was at 1550 °C, the emission intensity of sample with x = 0 was the strongest one relative to other Ga^3+^ concentrations of the samples; x = 1 was strongest at 1450 °C, and x = 2 was strongest at 1350 °C.

This phenomenon is consistent with the analysis on the crystal structure and micro-morphology. Generally, after exceeding the phase-forming temperatures, the emission intensity becomes stronger with the increasing particle size and sintering temperature. In general, the defect-free particles of phosphors with spherical or nearly spherical shape exhibit a superior emission intensity. The emission intensity of the as-prepared phosphors reached its maximum at the just phase forming temperature. Subsequently, with the increase in Ga^3+^ ion concentration, the phase formation temperature point decreases, resulting in the increase in particle aggregation at the same sintering temperature. Finally, it causes the emission intensities to decrease gradually with the increase in the synthesis temperature.

As shown in Figure 4a–c, the PL spectra of the Lu_3_Ga_x_Al_5−x_O_12_: Ce^3+^ phosphors excited at 450 nm displayed a gradual blue-shift with the increasing Ga^3+^ content. Although similar phenomena have also been found in previous studies of the luminescent properties of YAG: Ce^3+^ [13,14], few have been systematically researched for the effect of Ga^3+^ doping on the luminescent properties in LuAG: Ce^3+^ at different temperatures. As exhibited in Figure 4b, the variety of peak position in the emission spectra display from 508 nm to 482 nm was accompanied by the change of x from 0 to 3. The blue-shift offset was 26 nm. Based on the above results, it can be predicted that a series of Ga^3+^-substituted lutetium aluminum garnet will be excellent stuffing into the blue-green cavity of the emission spectrum around 490 nm, which is conducive to a high CRI. Furthermore, the full-width at half-maximum (FWHM) of the emission profile with the introduction of Ga^3+^ ions broadened a small amount from 75 nm (x = 0) to 79 nm (x = 3), which will also be helpful in obtaining a high quality illumination source in w-LED applications.

As illustrated in Figure 4d, monitored at the optimal emission, all of the PLE spectra with similar morphological characteristics included two broad absorption bands. These two broad bands, peaking around 350 nm and 430 nm, were assigned to the 4f→5d2,1 spin-allowed transition of the Ce^3+^ ions. By comparing these curves, it can be found that with the increase in the Ga^3+^ ion concentration, the higher energy excitation band (4f-5d2) and the lower energy excitation band (4f-5d1) moved toward each other gradually. This phenomenon for the spectral shift of PL and PLE can be determined by two possible factors: the crystal field splitting (CFS) and the nephelauxetic effect (NE) [13,21,22].

According to reports by Robertson et al., the degree of crystal field splitting (*D*_q_) can be defined as follows [23]:

(1) $$D_{q}=\frac{1}{6}Ze^{2}\frac{r^{4}}{R^{5}}$$

where *D*_q_ represents the degree of energy level separation; *Z* is the charge of anion; *e* is the charge of electron; *r* is the radius of the *d* wavelength; and *R* is the bond length between the activator and coordination ion. As we know, a garnet-type structure with the chemical formula A_3_B_2_C_3_O_12_ consists of three different structures: AO_8_ (distorted dodecahedral), BO_6_ (octahedral), and CO_4_ (tetrahedral) framework. Ascribed to the larger cationic radius of Ga^3+^ ions than Al^3+^ ions, the cell parameters and cell volume (V) will increase with the replacement of Al^3+^ by Ga^3+^ at the tetrahedral and octahedral sites. This means that the bond distance (*R*_Ce−O_) will become longer when Ce^3+^ ions enter the Lu^3+^ site. It can be inferred from the inverse proportional formula between *D*_q_ and *R* that the decrease in the crystal field intensity will cause the emission wavelength to move to the high-energy region, resulting in a continuous blue-shift of the PL spectra.

In addition, the positive correlation exists between the centroid shift (ε_c_) of the Ce^3+^ 5d levels and the anion polarizability (α_sp_) based on the reports of Morrison and Dorenbos [19,20]. The anion polarizability in Ce^3+^ doped oxide compounds can be estimated by the following equation [24,25],

(2) $$\alpha_{sp}=0.33+\frac{4.7}{\chi_{av}^{2}}$$

Meanwhile, the *α_sp_* is affected by the joint action of the average electronegativity *χ_av_* of the cations in the host:

(3) $$\chi_{\mathrm{av}}=\frac{1}{N_{a}}\sum_{1}^{N_{c}}\frac{z_{i}\chi_{i}}{\gamma_{i}}$$

where *χ_i_* denotes the electronegativity of cation *i* with formal charge *z_i_*; *N_c_* represents the summation over all cations in the compound; *N_a_* and *γ_i_* stand for the number and the formal negative charge of the anion in the formula, respectively. By Plugging Pauling-type electronegativity values in Equation (3), the values of χ_av_ were calculated to be 1.483, 1.508, 1.533, 1.558, and 1.583 in Lu_2.94_Ga_x_Al_5−x_O_12_: 0.06Ce^3+^ for x = 0, 1, 2, 3, and 4, respectively. Based on Equation (2), the increasing χ_av_ will lead to the decrease in the anion polarizability α_sp_. As a result, the centroid of the Ce^3+^ 5d levels shift to a higher-energy position, resulting in a gradual blue-shift of the emission and excitation bands with the increase in the Ga^3+^ ion concentration from 0 to 4.

Figure 4d also shows that the 4f–5d1 excitation band of Lu_2.94_Ga_3_Al_2_O_12_: 0.06Ce^3+^ exhibited a larger blue-shift (20 nm), while the 4f–5d2 excitation band showed a smaller redshift (9 nm) compared to the excitation spectral band of Lu_2.94_Al_5_O_12_: 0.06Ce^3+^. Similar phenomena have been found in other research, but have been rarely mentioned [11,19]. This phenomenon of spectral shift caused by Ga^3+^ doping in Re_3_Al_5_O_12_:Ce^3+^ host (Re = Lu, Y) can be explained by the combined effects of CFS and NE. The experimental results combined with previous studies are listed in Table 1 [13,18,19,20]. It can be seen that a larger shift (represented by ↑↑) of the excitation band, which was attributed to the elevated energy level, was co-promoted by CFS and NE. The contradictory role of CFS and NE on the energy level led to a slighter shift (represented by ↓) of the excitation band. Meanwhile, this result also illustrates that CFS plays a more significant role than that of the increase in NE in the phosphors that are activated by Ce^3+^.

**Figure 5:**
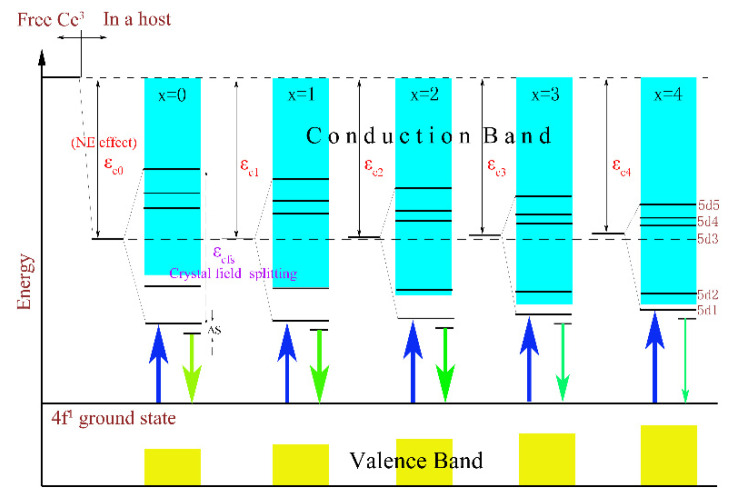
The effects of CFS and NE on the energy levels of the Lu_2.94_Ga_x_Al_5−x_O_12_: 0.06Ce^3+^ (x = 0, 1, 2, 3, 4) phosphors and its ionization processes.

In addition, a clear evolutionary trend could also be observed in Figure 4 where the PL and PLE intensities of the samples with the increase in x gradually weakened and even emitted no radiation (as x = 4). The thermoelectric ionization model can be used to explain this phenomenon. According to previous studies [26], the valence band energy E_V_ rose from −9.6 eV for Lu_3_Al_5_O_12_ to −9.0 eV for Lu_3_Ga_5_O_12_, but the conduction band energy E_C_ declined from −1.7 eV to −2.5 eV. As a result, with the increase in the Ga^3+^ ion content, the 5d1 level will gradually approach the conduction band, and the 5d2 level will even enter the conduction band. Namely, the energy gap between the conduction band and the lowest energy of the 5d excited state of the host lattice will be reduced. This result enhances the probability of thermal ionization, and ultimately leads to a reduction in the emission intensity. The schematic diagram is described in Figure 5.

In summary, the luminescence intensity of samples was determined by the synthesis temperature and energy gap. For one thing, the increasing sintering temperature will result in the aggregation of particles in Lu_3_Ga_x_Al_5−x_O_12_: Ce^3+^, which will worsen the emission strength after grinding; for another, the energy gap between the conduction band and the lowest energy of the 5d excited state of host lattice is reduced with the increase in the Ga^3+^ concentration as it forces enormous excited electrons into entering the conduction band and leads to the poor or even non-luminous intensity.

### 3.3. Temperature Dependence Luminescence Intensity

**Figure 6:**
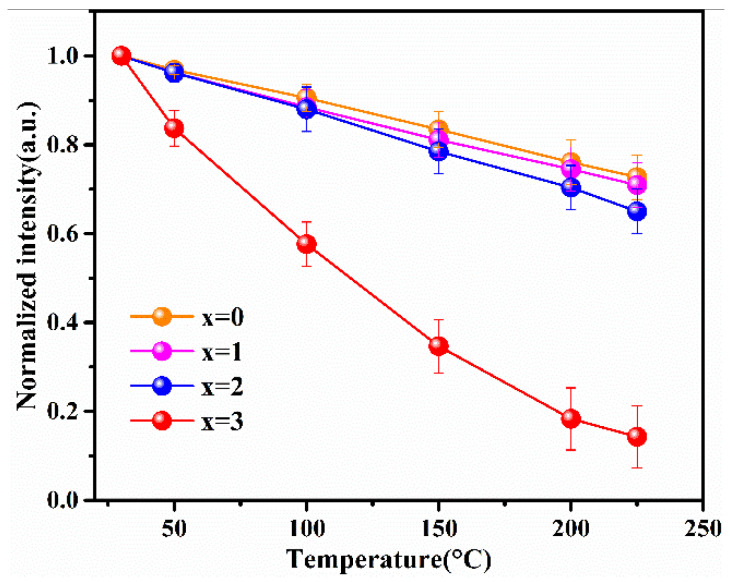
The thermal quenching behavior in the Lu_3_Ga_x_Al_5−x_O_12_: Ce^3+^ (x = 0, 1, 2, 3) phosphors sintered at 1450 °C.

When w-LEDs are in service, ambient temperatures of phosphors can typically reach 150 °C. In order to ensure the high energy efficiency and color stability of w-LEDs, the excellent thermal stability of phosphor is a crucial performance parameter. The temperature dependence of the normalized emission intensity for the Lu_2.94_Ga_x_Al_5−x_O_12_: Ce^3+^_0.06_ (x = 0, 1, 2, 3) phosphors at T = 30–225 °C are shown in Figure 6. Lu_2.94_Ga_4_AlO_12_: Ce^3+^_0.06_ is not shown in the figure as it had almost no luminous properties. Statistical error of the thermal quenching measurement equipment was estimated as error bars.

**Figure 7:**
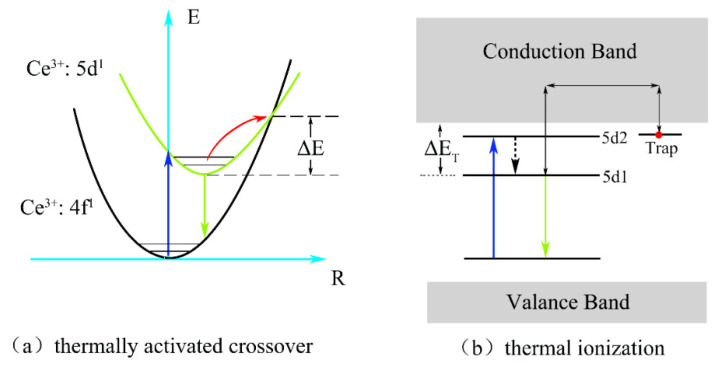
(**a**) The thermally activated crossover and (**b**) thermal ionization model for the thermal quenching.

As can be observed in the figure, the integrated normalized emission intensity of the phosphors exhibited a quite monotonic decrease with the increase in the ambient temperature. Meanwhile, as the Ga^3+^ ion concentration increased, the normalized emission intensity tended to decrease, indicating that the thermal stability gradually deteriorated. It can also be observed that when the temperature was measured at 150 °C, the emission intensities of these samples dropped to 83%, 81%, and 79% of the initial value (25 °C), demonstrating an excellent stability against the thermal quenching of x = 0, 1, and 2, respectively. However, the emission intensity declined sharply as x = 3 with the rising temperature. The reason for this phenomenon is the gradual intensification of the interaction between the phonons and electrons with increasing temperature, leading to an increase in the probability of non-radiative transition. As a widely accepted theory, the thermally activated crossover mechanism is used to explain the weakening in thermal stability of Ga^3+^-doped phosphor. As shown in Figure 7a, excited electrons with sufficient thermal activation energy(ΔE) can pass through the cross-relaxation point between the 5d1 level and 4f level, and return relatively easily to the ground state with the increasing temperature in a non-radiative relaxation manner. However, according to the spectral analysis results, with the increase in the Ga^3+^ ion concentration, the blue-shift phenomenon of the emission spectrum indicates that the Stokes shift will decrease, which will lead to the increase in the ΔE value. In general, the thermal stability of phosphor with a larger value of ΔE will improve rather than deteriorate. However, this theory and its conjectures are contrary to the experimental results of thermal quenching. Herein, it can be inferred that the thermally activated crossover mechanism is not the dominant factor influenced on the thermal quenching of the system. Therefore, the thermal ionization mechanism was used to elaborate this abnormal thermal quenching phenomenon.

As shown in Figure 7b, the energy gap (ΔE_T_) for the thermal ionization process, which is between the conduction band and the lowest energy of the 5d excited state, will narrow with the increase in the Ga^3+^ ion concentration. It can be inferred that the probability of 5d electrons enter into the conduction band based on thermal fluctuation will become more obvious with the increase in temperature. Hence, compared to the thermally activated crossover process, thermal ionization is more likely to cause non-radiative transition of 5d1 level electrons in the excited state and explains the reason quite well as to why there was a sudden deterioration phenomenon of thermal stability from x = 2 to 3. Therefore, the thermal ionization process, instead of the thermally activated crossover process, becomes the main reason for the strong thermal quenching for Lu_2.94_Ga_x_Al_5−x_O_12_: Ce^3+^_0.06_ with the increase in the Ga^3+^ content.

### 3.4. Packaging Test

**Figure 8:**
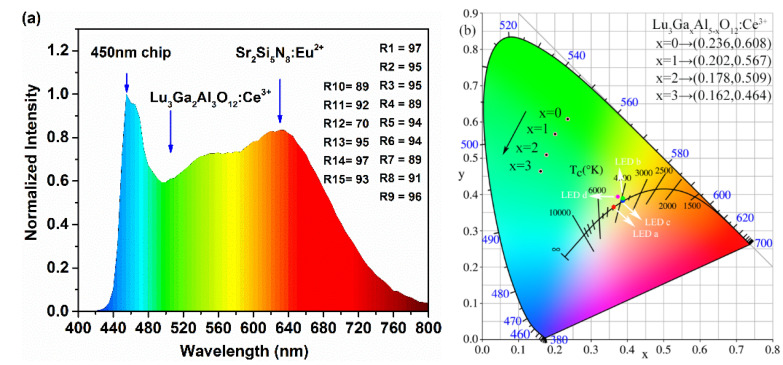
(**a**) The luminescence spectrum of the specimen w-LED device. (**b**) The CIE chromaticity coordinates of the Lu_3_Ga_x_Al_5−x_O_12_: Ce^3+^ (x = 0, 1, 2, 3) phosphors and specimen LEDs a, b, c, and d.

Aiming to evaluate the effectiveness of the cyan-green phosphor LuAGG: Ce^3+^(x = 0, 1, 2, 3) in practical application, a specimen of the w-LED device was obtained by mixing the as-prepared Lu_2.94_Ga_2_Al_3_O_12_: Ce^3+^_0.06_ sample with a certain amount of commercial red phosphor Sr_2_Si_5_N_8_: Eu^2+^ and encapsulating them on the blue GaN chip (450 nm). The emission spectrum of the as-fabricated w-LED device driven by a current of 20 mA is present in Figure 8a. Obviously, compared with the YAG: Ce^3+^ and LuAG: Ce^3+^ spectra [11,25], it can be conspicuously observed that the blue-green cavity of the emission spectrum of the as-prepared phosphor was filled and the spectrum covered the whole visible region smoothly.

The experimental LED parameters of the specimen LED device marked as a, b c, and d including the CIE chromaticity coordinates, CRI, CCT, and luminous efficiency are listed in Table 2 and drawn in Figure 8b.

The results in Table 2 show that with the increase in the Ga^3+^ content in the as-prepared cyan-green phosphor, the CRI of the specimen LED device increased at first, and then dropped, which reached its maximum value at x = 2; the color temperature and the light efficiency decreased gradually. The reason for the increase in the CRI value is that the blue-green cavity of the emission spectrum was filled by an appropriate substitution of Ga^3+^ for Al^3+^, which could significantly improve the color index properties of the white LED device. However, due to the significant reduction in the emission intensity of the samples and the spectral absorption of red phosphors to the samples, these may be the reasons as to why the luminous efficiency of the w-LED device decreased when x = 3.

The CIE chromaticity diagram of these samples is drawn in Figure 8b, which demonstrates a succession of transitions from x = 0.236, y = 0.608 (green region) to x = 0.162, y = 0.464 (cyan region) with the Al^3+^ sites gradually occupied by Ga^3+^ ions. The results indicate that the as-prepared phosphors are hopeful in making a tunable blue-green emission under the excitation of blue light in order to compensate for the lack of spectrum in the 470–510 nm region.

## 4. Conclusions

A series of Lu_2.94_Ga_x_Al_5−x_O_12_: Ce^3+^_0.06_ phosphors was synthesized in a nonreducing atmosphere by the high temperature solid state method at different temperatures. The phase formation temperature of these phosphors decreased with the increase in the Ga^3+^ concentration. The luminescence intensities of these samples were affected by the synthesis temperature and energy gap (ΔE_T_). The peak position of emission spectrum under λ_ex_ = 450 nm revealed a blue-shift from 508 nm to 482 nm based on the joint action of the crystal field splitting of 5d energy levels and nephelauxetic effect. Furthermore, the PL intensity of the samples (x = 1 and 2) still remained at 81% and 79% of the initial values (25 °C), which compared favorably with LuAG: Ce^3+^. The main reason for thermal quenching was elucidated by the thermal ionization model rather than the thermally activated crossover process. Meanwhile, by assembling the Lu_2.94_Ga_2_Al_3_O_12_: Ce^3+^_0.06_ phosphor and commercial red phosphor Sr_2_Si_5_N_8_: Eu^2+^ on a blue LED chip (450 nm), the blue-green cavity in the luminescence spectrum of the specimen LED can almost be filled. Finally, a satisfactory combination of parameters including a CCT of 3880 K and a CRI of 93.2 was obtained. The results demonstrate that the Lu_2.94_Ga_x_Al_5−x_O_12_: Ce^3+^_0.06_ phosphors synthesized through a nonreducing atmosphere possess great potential for application in w-LEDs with high CRI.

## Figures and Tables

**Table 1:** The relationship between CFS/NE and energy levels of LuAG: Ce^3+^, Lu_3_(Mg,Si)_3_Al_2_O_12_: Ce^3+^, Lu_3_Ga_3_Al_2_O_12_: Ce^3+^, YAG: Ce^3+^, and Y_3_Ga_3_Al_2_O_12_: Ce^3+^ phosphors. “0” represents the initial position of energy levels, “↑” and “↓” represent the increase and decrease of energy levels, respectively.

Sample	χ_av_	NE	CFS		Combined Effects		Ref.
			5d2	5d1	5d2	5d1	
Lu_3_Al_5_O_12_: Ce^3+^	1.480	0	0	0	0	0	[13,18,19,20]
Lu_3_(Mg, Si)_1.5_Al_2_O_12_: Ce^3+^	1.538	↑	↑	↓	↑↑	↓	[18]
Lu_3_Ga_3_Al_2_O_12_: Ce^3+^	1.558	↑	↓	↑	↓	↑↑	This work
Y_3_Al_5_O_12_: Ce^3+^	1.464	0	0	0	0	0	[13,20]
Y_3_Ga_3_Al_2_O_12_: Ce^3+^	1.539	↑	↓	↑	↓	↑↑	[20]

**Table 2:** The LuAGG: Ce^3+^ (x = 0, 1, 2, 3) test sample with white LED parameters.

Specimen LED	Sample	x	y	CCT (K)	CRI	Luminescence Efficiency (lm/W)
a	x = 0	0.361	0.367	4531	89.5	41
b	x = 1	0.387	0.388	3924	92.3	38
c	x = 2	0.387	0.384	3880	93.2	36
d	x = 3	0.373	0.392	3613	82.3	31

## Data Availability

Not applicable.
